# Evaluation of Durability as a Function of Fabric Strength and Residual Bio-Efficacy for the Olyset Plus and Interceptor G2 LLINs after 3 Years of Field Use in Tanzania

**DOI:** 10.3390/tropicalmed8080379

**Published:** 2023-07-25

**Authors:** Salum Azizi, Jackline Martin, Njelembo J. Mbewe, Agness Msapalla, Silvia Mwacha, Amandus Joram, Benson Mawa, Robert Diotrephes Kaaya, Jovin Kitau, Franklin Mosha, Johnson Matowo, Natacha Protopopoff

**Affiliations:** 1 Department of Medical Parasitology and Entomology, Kilimanjaro Christian Medical University College, Moshi 255, Tanzaniasilvia.mwacha@pamverc.or.tz (S.M.); robert.kaaya@kcmuco.ac.tz (R.D.K.); fwmosha@pamverc.or.tz (F.M.); johnson.matowo@kcmuco.ac.tz (J.M.); 2 Pan African Malaria Vector Research Consortium, Moshi 255, Tanzanianatacha.protopopoff@lshtm.ac.uk (N.P.); 3 Department of Disease Control, London School of Hygiene and Tropical Medicine, London WC1E 7HT, UK; 4 Country Office, World Health Organization, Dar es Salaam 255, Tanzania; kitauj@who.int

**Keywords:** long-lasting insecticidal nets, fabric strength, bio-efficacy, fabric damage, chemical and physical protection

## Abstract

Long-lasting insecticidal nets (LLINs) are prone to reduction in insecticide content and physical strength due to repeated washes and usage. The significant loss to these features jeopardizes their protection against bites from malaria vectors. Insecticide washout is attributed to routine use, friction, and washing, while fabric damage is associated with routine use in households. To maintain coverage and cost-effectiveness, nets should maintain optimal bio-efficacy and physical strength for at least 3 years after distribution. In this study, the bio-efficacy and fabric strength of Olyset plus (OP) LLINs and Interceptor G2 (IG2), that were used for 3 years, were assessed in comparison to untreated and new unwashed counterparts. Both IG2 and OP LLINs (unused, laboratory-washed, and 36 months used) were able to induce significant mortality and blood feeding inhibition (BFI) to mosquitoes compared to the untreated nets. Significantly higher mortality was induced by unused IG2 LLIN and OP LLIN compared to their 36-month-old counterparts against both pyrethroid resistant and susceptible *Anopheles gambiae* sensu strito. The physical strength of the IG2 LLIN was higher than that of the Olyset Plus LLIN with a decreasing trend from unwashed, laboratory-washed to community usage (36 months old). Malaria control programs should consider bio-efficacy and physical integrity prior to an LLINs’ procurement and replacement plan.

## 1. Introduction

Long-lasting insecticidal nets (LLINs) are the main vector control tools for malaria prevention, and are responsible for significant malaria reduction in sub-Saharan Africa over the past two decades [1,2]. Between 2000 and 2019, WHO reported 1.5 billion malaria cases and 7.6 million deaths being averted [3]. The LLINs provide personal protection by preventing human–vector contact through a physical barrier [4], and providing a killing effect, repelling, sterilizing or inhibiting blood feeding through their insecticide component [5]. It is evident that the LLIN maintains its bio-efficacy (chemical protection) even when the LLIN is physically damaged [5]. Consequently, LLINs have been regarded as a main vector control tool against malaria vectors [6,7]. As a result, more than 1.9 billion Insecticide-treated nets (ITNs) were deployed in sub-Saharan Africa between 2004 and 2019 [3]. However, malaria reduction has stalled in the past few years [8] due, in part, to widespread resistance in malaria vectors to pyrethroid the insecticides, the only class of insecticides used for bed net treatments. Novel insecticide-treated nets have been developed to control resistant vectors [9] and two of those nets have demonstrated superior efficacy on entomological and malaria indicators compared to standard pyrethroid LLINs [10,11], and hence are recommended by the WHO for deployment [12]. One of them combines a pyrethroid and a synergist piperonyl butoxide (PBO) which enhance the killing property of the insecticide and the second combines a pyrethroid and a second insecticide chlorfenapyr, which has a different mode of action, disrupting the cellular respiration of the insect rather than affecting its nervous system. 

The LLINs are supposed to preserve their insecticidal activity and physical integrity for 20 standard washes in a laboratory setting and at least 3 years of usage in the field, as recommended by the World Health Organization (WHO) [6]. The standard assessment of long lasting bio-efficacy is carried out by exposing susceptible and resistant *Anopheles,* using WHO cone assays or tunnel tests, on net pieces either washed in the laboratory for phase I or collected from the community every 6 months in phase III. According to recent findings [13,14,15,16], most LLINs do not meet the WHO criteria in the community and last for only 2 years or below, due to insecticidal decay or holes formation (poor physical integrity), hence subjecting the users to a high risk of malaria transmission. Generating this evidence for novel insecticide-treated net is particularly important since there is limited information regarding the long-lasting bio-efficacy of LLINs after field use, despite the fact that the LLINs are massively distributed in most Sub-Saharan African countries [3].

While laboratory testing is an integral part of assessing LLIN’s insecticidal bio-efficacy, much less consideration has been paid to mechanical damage, which accounts for the majority of holes that develop in LLINs [17]. The bursting strength test, which, for years, has been the only test for physical integrity prior to distribution in the field, is known to be a poor predictor of the likelihood of LLINs developing holes during daily normal usage [2]. Therefore, more approaches for the physical strength assessment of LLIN performance are needed, including the fabric strength [18], targeting most common damage mechanisms that LLINs encounter during real use conditions [19]. The recommended predictive tests of LLIN durability, namely pneumonic bursting strength, wounded bursting strength, grab tensile and hook tensile methods can closely predict the likelihood of LLIN to develop holes during daily usage. Premature damages, which often occur in a shorter timeframe than the interval between replenishment campaigns, are attributed to shortened lifespan and low usage of LLINs. Although new insecticide-treated nets containing pyrethroid and PBO, such as Olyset Plus, or pyrethroid and non-pyrethroid, such as Interceptor G2 (IG2), have shown a greater reduction in the incidence of malaria than pyrethroid-only LLINs in areas with pyrethroid-resistant mosquitoes [10,20,21,22,23,24], their durability, especially their physical integrity under operational settings, has not been fully characterized. 

Given the importance of both physical and chemical protection for the LLIN, the purpose of the current study was to determine how IG2 and Olyset Plus LLINs would maintain fabric strength and bio-efficacy after being subjected to simulated washes in the laboratory compared to realistic community uses.

## 2. Materials and Methods

### 2.1. Study Site

The WHO cone bioassays [25] for bio-efficacy were conducted at the KCMUCo-PAMVERC Insecticide Testing Facility. The 36-month-old LLINs used in this study were among the study nets of previous study [20], that was conducted in Misungwi District located on the southern border of Lake Victoria, northwestern Tanzania (2°51′00.0″ S, 33°04′60.0″ E).

The fabric integrity tests were conducted at the Technological Centre for the Textile and Clothing Industry of Portugal, CITEVE (Centro Tecnológico Industrias Têxtil Vestuário, Vila Nova de Famalicão, Portugal).

### 2.2. Test Items (Nets Used in the Study)

The test items included the polyester IG2 and the polyethylene Olyset Plus LLINs. These nets were retrieved from the previous distribution by replacement at the end of the third year of use during the project duration. Interceptor G2 is a multifilament polyester net produced by BASF SE using a proprietary polymer system. It contains 200 mg/m^2^ chlorfenapyr and 100 mg/m^2^ alpha-cypermethrin. Olyset™ Plus, on the other hand, is a long-lasting insecticidal net (LLIN) containing 2% of the pyrethroid insecticide permethrin and 1% of the synergist piperonyl butoxide (PBO), developed by Sumitomo Chemical. More specifications for Olyset Plus and IG2 are provided in Table 1. 

Sampling and sample size: For each net type, we randomly selected four new and unwashed nets; all the nets belonged to the same purchase batch as the nets distributed in the community. Two of the new nets were consequently washed 20 times in laboratory conditions following the WHO guidelines [25]. One of the washed nets was dedicated for bio-efficacy and the second one for fabric integrity. Similarly, one of the unwashed new was dedicated for the bio-efficacy and a second one for fabric integrity tests. From the 36-month-retrieved nets, 2 nets were randomly selected from each type (IG2 and Olyset Plus). From each type, one net was dedicated for bio-efficacy and the second one was used for fabric integrity tests. Although there might be some variation between nets of the same type, since all these nets were from the same purchase batch, we assumed less or no variation between the same net types. Nets of the same type from the same procurement order are more homogenous and are expected to comply with manufacturer’s specifications in terms of active ingredient and fabric properties. Furthermore, manufacturers have claimed that their product fulfils the WHO specification, IG2 (454/TC and 570/TC for alpha-cypermethrin and chlorfenapyr, respectively) and Olyset Plus. The following treatments were used in both bio-efficacy and physical strength tests:

1. New unused Olyset Plus nets (1 for bio-efficacy, 1 for physical integrity)
2. New unused Interceptor G2 nets (1 for bio-efficacy, 1 for physical integrity)
3. Laboratory-washed Olyset Plus nets (1 for bio-efficacy, 1 for physical integrity)
4. Laboratory-washed Interceptor G2 nets (1 for bio-efficacy, 1 for physical integrity)
5. Interceptor G2 nets used under operational conditions for 36 months (1 for bio-efficacy, 1 for physical integrity)
6. Olyset Plus nets used under operational conditions for 36 months (1 for bio-efficacy, 1 for physical integrity)

### 2.3. Net Washing

To prepare net samples, which were washed 20 times, one IG2 and Olyset Plus nets were cut into four, 25 × 25 cm pieces and then two pieces were washed and dried twenty times following WHO guidelines [25]. Time between two washes was 1 day for IG2 [21] and 2 days for Olyset plus [10] following previously established regeneration time.

### 2.4. Cone Bioassays

Five, non-blood fed, 2–5-day old *An. gambiae* Kisumu or *An. gambiae* Muleba-Kis mosquitoes were exposed for 3 min to net pieces in cone WHO bioassays under controlled environmental conditions of 27 ± 2 °C and 75 ± 10% RH; set-up and procedures were as per the WHO guidelines [25]. Different treatments were held in separate panels (with each having capacity to hold 4 net pieces with cones) to avoid cross-contamination. A total of 5 mosquitoes per cone were used on each piece of net sample and experiment was replicated 8 times (40 mosquitoes exposed to each piece). Each treatment had two pieces. After exposure, the mosquitoes were held for 72 h with access to 10% glucose solution in the net-covered paper cups. Outcome in terms of knock-down was scored after 60 min post-exposure and mortality after 24, 48, and 72 h.

### 2.5. WHO Tunnels Test

Net pieces which failed (≤80% mortality) in cone bioassay were tested in the tunnel bioassay following WHO guidelines [25]. The tunnels are made of an acrylic square cylinder (25 cm in height, 25 cm in width, and 60 cm in length) with a bait chamber separated using a net piece fitted into a slot across the tunnel. The net piece was intentionally holed (nine 1 cm holes) to allow mosquitoes to contact the net material and penetrate the baited chamber. During the bioassay, a single guinea pig (as a bait) was introduced in bait chamber and 50, non-blood fed, female *An. gambiae* Kisumu or *An. gambiae* Muleba-Kis mosquitoes, aged 5–8 days, were released in the other section at dusk and left overnight (13 h) under environmental conditions of 27 ± 2 °C and 75 ± 10% RH. Treated nets (one piece of IG2 unwashed, IG2 washed, IG2 community used, OP unwashed, OP washed, and OP community used) were tested along with an untreated net. Two replicates were run, making a total of 100 mosquitoes exposed to each net piece. Scoring for the numbers of mosquitoes found alive or dead, fed or unfed, in each section was carried out in the morning. Mosquitoes found alive were transferred to paper cups with labels corresponding to each tunnel sections and held under controlled conditions (25–27 °C and 75–85% RH) with access to 10% glucose solution. Outcomes recorded were: mosquito penetration, blood feeding, and mortality.

### 2.6. Nets Fabric Integrity

All tests were conducted at the Centro Tecnologico das Industrias Textil e do Vestuario (CITEVE) de Portugal laboratories in Portugal. Single whole nets representative of all treatments (IG2 unwashed, IG2 washed, IG2 community used, OP unwashed, OP washed, and OP community used) were tested for fabric weight, tear strength, bursting strength, mesh size test, and modified and conventional tensile strength tests following the recommended WHO set-up and methods for fabric strength [2]. These tests measured the level of resistance to mechanical damage against simulated mechanisms that lead to LLINs damage during use in households. Snagging (most frequent cause of net damage during use, as a consequence of LLINs being caught on sharp or solid objects), tearing, and hole expansion are forms of damage affiliated with the specification of the LLIN fabric and the characteristics of its constituent polymer and yarn elements. Physical strength testing is therefore an integral part of ensuring LLINs meet quality specifications for fabric integrity.

### 2.7. Fabric Weight

Method EN 12127:1997 [2] was followed. In brief, 5 specimens were cut from each net into a 100 cm^2^ circular specimen. Cut specimens were weighed on a balance and the result multiplied by 100 to give results in g/m^2^. All the tests were conducted under conditioned atmosphere of 20 ± 2 ℃ and 65 ± 4% R.H.

### 2.8. Tear Strength Test (Ballistic Pendulum)

Method EN ISO 13937-1:2000+AC: 2006 [2] was followed at the CITEVE laboratory, Portugal. In brief, 5 specimens were cut from a 20 mm slit, at which a tear was initiated to produce a tear that terminated at the 15 mm notch on the opposite end of the sample. Measuring range used: length (32 N) and width (32 N). There were 5 specimens in both directions.

### 2.9. Bursting Strength

Method EN ISO 13938-2:2019 [2] was followed. In brief, a pneumatic testing apparatus, Tru-Burst (James Heal, PPT Group Corp, Virginia, USA) was used. Air pressure was applied to the side of the diaphragm opposite the net specimen, and the diaphragm was forced against the specimen. The pressure was increased until the specimen ultimately burst; the pressure and diaphragm distension were recorded (in kPa) at the moment of bursting. There were 5 tested specimens under conditioned test, with tested area of 7.3 cm^2^.

### 2.10. Mesh Size Test

The CITEVE Method 110 of a modified FAO/WHO method [18] was followed. In brief, 5 specimens were measured for mesh size from each net, and the average number of complete holes per unit area were recorded (holes/cm^2^). Before counting, the fabric was conditioned according to ISO 139 (4 h, 20 °C, 65% relative humidity).

### 2.11. Tensile Strength Test (Hook Tensile Strength Test)

Method ISO 13934-2:2014 Modified, “standard grab and modified using hooks” [2], was followed at the CITEVE laboratory, Portugal. In brief, maximum force required to rupture a 100 mm specimen fixed in clamps (with metal hooks that pass through the netting mesh), set 100 mm apart and elongated uniaxially at a rate of 50 mm/min, was measured and recorded in Newton (N).

### 2.12. Tensile Strength Tests (Standard Grab Method)

Method ISO 13934-2:2014, “standard grab” [2], was followed. In brief, maximum force required to rupture a 100 mm specimen fixed in clamps, set 100 mm apart and elongated uniaxially at a rate of 50 mm/min, was measured and recorded in Newton (N).

### 2.13. Statistical Methods

The number of mosquitoes per cones and tunnels were within the acceptable range of 5 ± 1 and 50 ± 2 mosquitoes, respectively. The control mortalities for cones bioassays and tunnel tests were all less than 10%, therefore, mortality in the insecticide treatment groups was not adjusted. For cone and tunnel test bioassays, Stata 16 (Stata Corp LP, College Station, TX, USA) was used to format the data, calculate all proportions of observations of interest (number knockdown/number dead) and the total observed, assuming α = 0.05. We have included a multiple logistic regression analysis and reported odds ratio in Supplementary Tables S1 and S2 that consider control mortality. In this case, the odds ratio represents the odds that an outcome (mortality) will occur given a particular exposure (test items), compared to the odds of the outcome occurring in the absence of that exposure (untreated nets). In this study, we have two scenarios of absence of exposure, i.e., untreated 0× washed and untreated 20× washed. The odds ratio in Supplementary Tables S1 and S2 are reported for both scenarios for each test item. The pairwise z-test for proportions was also performed for comparing proportions. The following formulas were applied:

$$Wilson\;confidence\;interval=\frac{p+\left(\frac{Z^{2}\alpha /2}{2n}\right)\pm Z\alpha /2\sqrt{\left(\frac{p\left(1-p\right)}{n}\right)+\left(\frac{Z^{2}\alpha /2}{4n^{2}}\right)}}{1+\left(\frac{Z^{2}\alpha /2}{n}\right)}$$

where:

*p* = a proportion;

*n* = the sample size in a trial arm;

*Z_α_*_/2_ = 1.96.

The BFI in the tunnel tests was calculated using formula:

$$Blood\;Feeding\;Inhibition=\left(\frac{BFc-BFt}{BFc}\right)\times 100$$

where:

*BFc* = number of mosquitoes blood fed in a control (untreated) tunnel;

*BFt* = number of mosquitoes blood fed in a treatment tunnel.

The penetration percentages in the tunnel tests were calculated using formula:

$$Penetration\;percentage=\left(\frac{BF+UFb}{N}\right)\times 100$$

where:

*BF* = total number of mosquitoes blood fed;

*UFb* = number of unfed mosquitoes found in a bait chamber;

*N* = total number exposed in a tunnel.

In this study, penetration is regarded as the number of mosquitoes that have crossed the net barrier.

Double entry, comparison checks, and accuracy checks on all the datasets were carried out in MS Access 2016. All datasets were transferred into Stata v16.0 (Stata Corp LLC, College Station, TX, USA) statistical software using Stat Transfer v8.0. All graphs were created in Microsoft Excel Office 2019 (Microsoft Corporation, Redmond, WA, USA) with proportions expressed as percentages.

For the net fabric strength, the expanded uncertainty presented was calculated by multiplying the standard uncertainty by the expansion factor k = 2 which, for a normal distribution, allows for associating the result at a confidence level of approximately 95%. For all the fabric strength measurements, relative expanded uncertainty was calculated using the formula:

Ur = U/|y|

where Ur = relative expanded uncertainty, U = expanded uncertainty, and y = measurement result (not equal to zero).

## 3. Results

### 3.1. Bio-Efficacy

#### 3.1.1. Cone Bioassays

**Figure 1:**
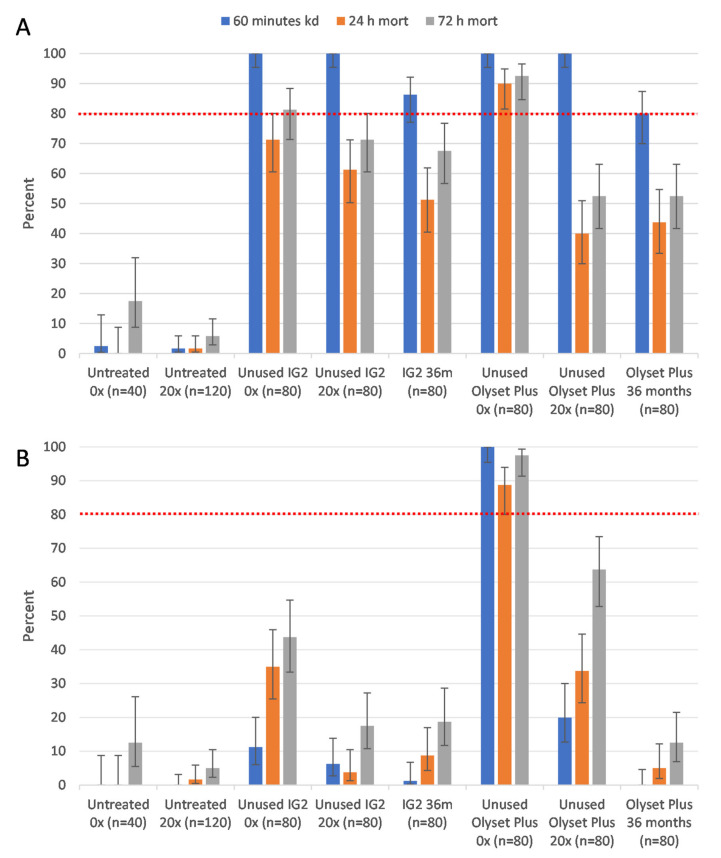
Induced mortality by IG2 and Olyset Plus LLINs in a cone bioassay against susceptible Kisumu (**A**) and resistant Muleba-Kis strain (**B**) of *An. gambiae* s.s. 0× = unwashed, 20× = 20 times washed in laboratory, and 36 m = 36-month-old net retrieved from community. n=total number of mosquitoes in each trial arm. Error bars represent 95% Wilson confidence intervals. Red dashed line is WHO 80% mortality threshold.

The results for cone bioassays (percentage mortalities) are summarized in Figure 1, while the pairwise comparisons of LLIN bio-efficacy are summarized as Supplementary Materials Tables S1 and S2.

All treated net pieces induced significant higher knockdown and mortality than the untreated negative control piece against susceptible *An. gambiae* Kisumu (Table S1), while a relatively lower performance was observed against the pyrethroid-resistant *An. gambiae* Muleba-Kis strain (Table S2). Furthermore, for the IG2, there was no significant difference in terms of mortality between the laboratory-washed net and its new unwashed counterpart (AOR = 0.84, CI: 0.43–1.65, *p* = 0.606). There was, however, a significant difference between the 36 month community-used net and new unwashed nets (AOR = 0.48, CI 0.23–0.99, *p* = 0.048). No significant difference was observed between the 36-month-old net and the laboratory-washed net (AOR = 0.57, CI: 0.27–1.20, *p* = 0.138) (Table S1). All Interceptor G2 net pieces reached and exceeded the WHO cut off (≥95%) with respect to 60 minutes knockdown post exposure at 100%, except the 36-month-old samples which were able to induce knockdown of 86.3% less than the WHO cut off. Contrarily, for the OP nets, both the 20-times-washed and 36-month-old community-used nets had significantly lower mortality compared to their new unwashed counterpart (0.09, CI: 0.03–0.23, *p* < 0.001) (Table S1). For knockdown, only the 36 month community nets were significantly lower (80%, CI: 69.9–87.3) than the WHO cut off value.

With respect to mortality at 24 h post exposure, mortality was 71.3%, 61.3%, and 51.3% for new unwashed, 20-times-washed, and 36-month-old samples from field use, respectively. However, with respect to mortality at 72 h post exposure, only the unwashed IG2 was able to induce a mortality of 81.3% (reaching the WHO cut off of ≥80%), which decreased to 71.3% when washed 20 times, and was 67.5% for the 36-month-old net collected from the community. 

Similarly, the unwashed Olyset Plus net pieces were able to induce knockdown (100%) and mortality 24 h post exposure (90%), both above the minimum WHO cut off, against Kisumu strain. However, mortality decreased rapidly with washes, where laboratory washes and community use induced mortality of only 48.8% and 40%, respectively, at 24 h post exposure. At 72 h post exposure, only the unwashed pieces were able to induce mortality above the WHO cut off (92.5%), which decreased to 52.5% when washed 20 times and 52.5% for the 36-month-old net collected from the community.

When tested against the pyrethroid-resistant Muleba-Kis strain, only the unwashed Olyset plus LLIN pieces were able to induce both knockdown (100%) and mortality (88.8%) above the WHO cut off. However, mortality was lower with the 20 washed samples (33.8%) and 36-month-old samples (5%). On the contrary, IG2 nets induced mortalities of 43.8%, 17.5%, and 18.8% when unwashed, 20 times washed, and 36 months old, respectively. The knockdown effect was extremely minimal, only reaching 1.3%, 6.3%, and 11.3% when 36 months old, 20 times washed, and new unwashed pieces were used. 

#### 3.1.2. Tunnel Test

##### Mortality Percentage

All treatments significantly induced higher mortality relative to the untreated control, against both the susceptible (Kisumu) and resistant (Muleba-Kis) mosquito strain. 

**Figure 2:**
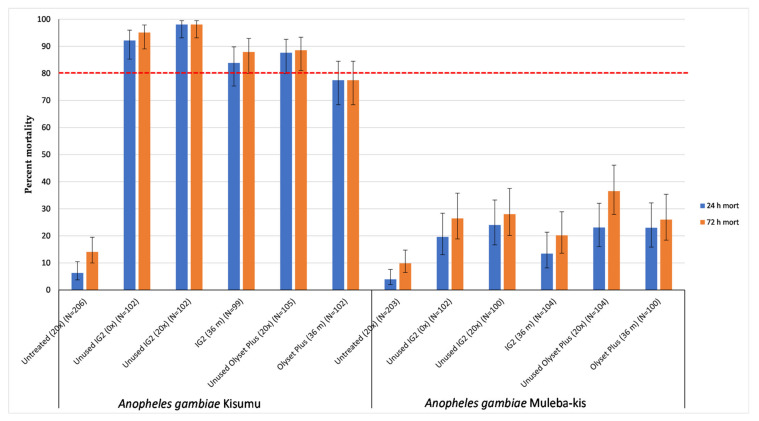
Tunnel test results for mortality induced to susceptible and resistant mosquitoes from nets subjected to different wash practices and frequencies. 0× = unwashed, 20× = 20 times washed in laboratory, and 36 m = 36-month-old net retrieved from community. N = total number of mosquitoes in each trial arm. Error bars represent 95% Wilson confidence intervals. Red dashed line is WHO 80% mortality threshold.

For the Olyset Plus, a mortality of 87.6% (above the WHO cut off of ≥80%) against the susceptible strain was observed when it was washed 20 times, with a relative lower mortality (77.5%) induced by the 36-month-old nets (Figure 2). However, when tested against the resistant strain, both laboratory-washed and 36-month-old net samples induced a lower mortality at 23.1% and 23%, respectively.

On the other hand, all samples from the IG2 induced mortality levels above the WHO cut off against the susceptible strain; 95.1% when unwashed, 98% when 20 times washed in the laboratory, and 87.9% in the 36-month-old net samples. However, when tested against the resistant strain, the unwashed samples, 20 times washed, and 36-month-old net samples induced lower mortalities (than the WHO cut off mortality) at 26.5%, 28%, and 20.2% respectively (Figure 2).

##### Blood Feeding Percentage

All treatments significantly reduced the blood feeding relative to the untreated control, against the susceptible Kisumu strain, but not against resistant (Muleba-Kis) mosquito strains. 

**Figure 3:**
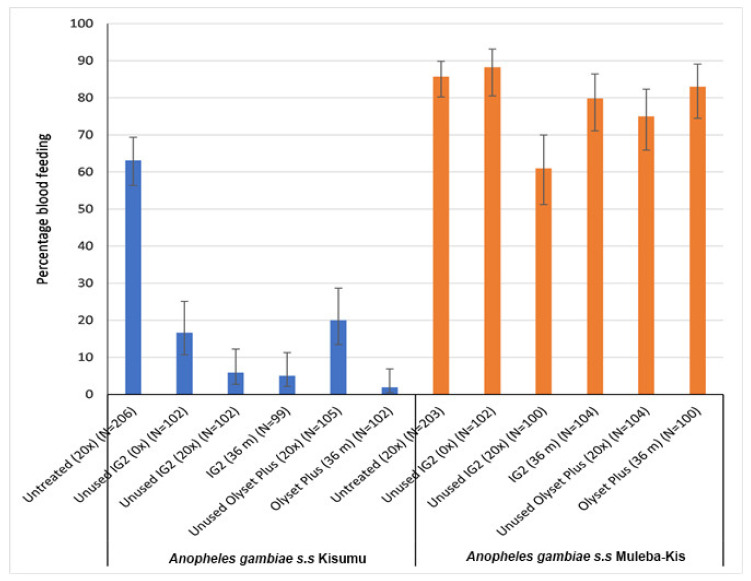
Tunnel test results for blood feeding reduction to susceptible and resistant mosquitoes from nets subjected to different wash practices and frequencies. 0× = unwashed, 20× = 20 times washed in laboratory, and 36 m = 36-month-old net retrieved from community. N=total number of mosquitoes in each trial arm. Error bars represent 95% Wilson confidence intervals.

For the Olyset Plus, blood feeding of 20% (equivalent to blood feeding inhibition (BFI) of 68.3%) against the susceptible strain was observed when it was washed 20 times, with a relative higher reduction in blood feeding ((2%) which was equivalent to 96.8% BFI), induced by the 36-month-old net samples (Figure 3). However, when tested against the resistant strain, both laboratory-washed and 36-month-old net samples did not indicate significant reductions in blood feeding compared to that of the untreated control net (85.7%).

Similarly, all samples from the IG2 significantly reduced blood feeding percentage compared to the untreated control net when tested against a susceptible strain: 16.7% when it was unwashed, 5.9% when washed in the laboratory (20 times), and 5.1% in the 36-month-old net samples. However, with the exception of a laboratory-washed sample which had a significant lower blood feeding (61%), when tested against the resistant strain, both the unwashed and 36-month-old net samples did not indicate significant reductions in blood feeding compared to that of the untreated control net (85.7%), as shown in Figure 3.

##### Penetration Percentage

With the exception of the IG2 samples washed 20 times, all treatments significantly reduced the percentage of penetration relative to the untreated control (68.4%) against the susceptible Kisumu strain, but presented a non-significant difference against the resistant (Muleba-Kis) mosquito strain. 

**Figure 4:**
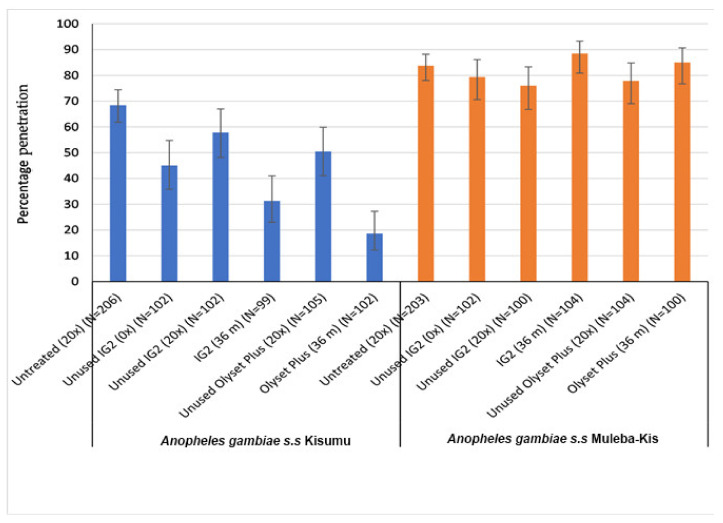
Tunnel test results for penetration rate by susceptible and resistant mosquitoes through nets subjected to different wash practices and frequencies. N = total number of mosquitoes in each trial arm. Error bars represent 95% Wilson confidence intervals.

For the Olyset Plus, a penetration of 50.5% against the susceptible strain was observed when it was washed 20 times, with a relative higher reduction in penetration (18.6%) induced by the 36-month-old net samples (Figure 4). However, when tested against the resistant strain, both the laboratory-washed and 36-month-old net samples did not indicate significant reductions in penetration percentage compared to that of the untreated control net (83.7%).

Similarly, with the exception of the IG2 samples washed 20 times (penetration of 57.8%), all samples from the IG2 significantly reduced penetration percentage relative to the untreated net control (68.4%) when tested against a susceptible strain: 45.1% by laboratory-washed and 31.3% by the 36-month-old net samples. However, when tested against the resistant strain, unwashed, laboratory-washed, and 36-month-old net samples did not indicate significant reductions in penetration compared to that of the untreated control net (83.7%), as indicated in Figure 4.

#### 3.1.3. Physical Strength 

##### Interceptor G2 LLIN

Pneumatic Bursting Strength

A high value (430 kPa) was observed for a new unwashed net. However, this decreased with washing and usage, where the laboratory-washed net resulted in 390 kPa and the 36-month-old net resulted in 376 kPa. Therefore, only the unwashed samples were within the manufacturer’s acceptable specifications for a 100 denier net (≥405 kPa). The 36-month-old net samples were the ones with a much reduced bursting strength.

Fabric Weight

This is an indirect measure of denier. The results showed no clear difference in mean fabric weight. The results for the new unwashed, laboratory-washed, and 36-month-old nets were 47.2, 45.8, and 48.2 g/m^2^, respectively. Nevertheless, all the values were within the WHO’s specifications [26]. 

Tensile Strength (Modified Grab Method with Hooks)

There was a slight decrease in mean tensile strength in both directions (length and width) with washing. Where the new unwashed net results were 19 Newtons (length-wise) and 24 Newtons (N) (width-wise), the 36-month-old nets were 18 N (length-wise) and 21 N (width-wise) while, for the laboratory-washed nets, they were slightly higher at 21 N (length-wise) and 26 N (width-wise). 

Tensile Strength (Standard Grab Method)

This is a measure of the tendency of fabric to rupture and snag and how the holes expand after initial snag. A decreasing trend in mean tensile strength was observed from new unwashed, laboratory-washed, to 36-month-old nets in both directions (length- and width-wise). Where the new unwashed net results were 224 N (length) and 148 N (width), the laboratory-washed results were 210 N (length) and 132 N (width), and the 36-month-old nets were 196 N (length) and 95.4 N (width).

Mesh size 

Only slight variations in average mesh size were observed without a clear trend with respect to wash/usage; the results for a new unwashed, laboratory-washed, and 36-month-old nets were 31, 29, and 30 holes/cm^2^. All values are within the specifications of the manufacturer and within the United Nations High Commissioner for Refugees (UNHCR) requirements (≥24 holes/cm^2^) [27].

Tear strength 

There was a decreasing trend in mean tear strength from new unwashed, laboratory-washed to 36-month-old net in both directions (length and width). 

##### Olyset Plus LLIN

Pneumatic bursting strength 

Only slight variations in average bursting strength were observed, without a clear trend with respect to wash/usage. Nevertheless, the bursting strength was within the specifications (±250 kPa) throughout.

Fabric weight

There was an increase in mean fabric weight from unwashed, laboratory-washed to 36-month-old nets. The results for the new unwashed, laboratory-washed, and 36-month-old nets were 38.7, 44.3, and 49.4 g/m^2^, respectively.

Tensile strength (modified grab method with hooks) 

Only slight variations in average tensile strength were observed without a clear trend with respect to wash/usage in both directions (length and width). The new unwashed net results had 19 N (length) and 21 N (width), while the results for the 36-month-old nets were 25 N (length) and 30 N (width). The tensile strength for the laboratory-washed nets was slightly higher at 18 N (length) and 23 N (width). 

Tensile strength (standard grab method) 

There was a decreasing trend in mean tensile strength from new unwashed, laboratory-washed to 36-month-old nets in both directions (length and width). 

Mesh size

There was an increasing trend in mean mesh size from new unwashed, laboratory-washed to 36-month-old nets with respect to wash/usage. The results for a new unwashed, laboratory-washed, and 36-month-old nets were 16, 21, and 22 holes/cm^2^, respectively. All values were within the specifications of the manufacturer (6.4 holes/cm^2^), but below the United Nations High Commissioner for Refugees (UNHCR) requirements (≥24 holes/cm^2^) [27].

Tear strength

There was no clear trend (increase/decrease) in mean tear strength from new unwashed, laboratory-washed to 36-month-old nets in both directions (length and width).

The results for fabric strength for Olyset Plus and Interceptor G2 are summarized in (Table S3).

## 4. Discussion

To fulfill their intended functions, LLINs must retain good physical condition in their fabrics and insecticide bio-efficacy during the whole duration of use [6]. Since the routine use of LLINs in the field is inevitably associated with primary sources of damage, it is therefore of paramount importance to assess the resistance of LLINs against damages.

This study evaluated the bio-efficacy and physical strength of Olyset® Plus (incorporated with permethrin and PBO) and Interceptor G2 (coated with alpha cypermethrin and chlorfenapyr), when used for 36 months in the field, compared to new and 20 times laboratory-washed counterpart nets. Their bio-efficacy was checked against *An. gambiae* Kisumu, a susceptible strain, and *An. gambiae* Muleba-Kis, a resistant strain [28]. A net was considered to have passed the WHO efficacy criteria if it induced ≥ 80% mortality and/or ≥ 95% knockdown in cone bioassays or ≥ 80% mortality and/or ≥ 90% blood-feeding inhibition in the tunnel tests [25]. New, Olyset ® Plus and Interceptor G2 LLINs recorded a full knockdown effect (100%) against susceptible *An. gambiae* s.s., regardless of whether they were washed or unwashed. After field use for 36 months, both Olyset ® Plus and Interceptor G2 nets maintained high knockdown effect but could not reach the WHO cut-off criteria (80% and 86.3%, respectively) in the cone bioassays. In terms of their mortality effect, the new unwashed Olyset Plus (90%) and Interceptor G2 (81.3%) met the criteria while, after 36 months of field use, the effect was lost by almost half in both Olyset Plus and Interceptor G2 nets. The pyrethroid content in new Olyset ® Plus and Interceptor G2 LLINs was 20g/kg and 2.4g/kg, respectively, way higher than the levels that would be required to knockdown a susceptible mosquito. The loss of the active ingredient on nets after repeated washing is expected. Losses of alphacypermethrin between 68 and 85%, and of permethrin between 46 and 67%, of the original content have been recorded after the repeated washing of Interceptor and Olyset ® Plus LLINs [20,29]. Washing frequency and practices in communities are closely linked to a loss of insecticidal content on nets; previous community studies attributed a high prevalence of unrecommended care of LLINs to a reduction in net durability in terms of bio-efficacy and fabric integrity [30]. When tested against a resistant strain, only the new unwashed Olyset Plus was able to induce knockdown (100%) and mortality (88.8%) over the WHO pass criterion. All other net treatments resulted into very low mortality and knockdown. In the tunnel tests, Interceptor G2 induced mortality above the WHO criterion, regardless of whether the nets were new, washed or 36 months old, at 95%, 98%, and 87.9%, respectively. On the other hand, the Olyset Plus was only able to induce mortality of 87.9% when it was washed, but its 36-month-old samples had a lower mortality at 77.5%. The improvement in the performance of IG2 in the tunnel connects to the mode of action of the active ingredient (chlorfenapyr), which is active during the increased metabolic activity of the vector, especially during the night time as per the tunnel test [31]. Again, short exposure assays like the 3-minute cone bioassay are ideal for pyrethroid-based LLINs, including Olyset Plus. For chlorfenapyr, a slow-acting pro-insecticide, a much longer exposure is needed. Evidence from previous studies [31] indicates that overnight experiments in tunnels increase mosquito activity and enhance the chlorfenapyr activity, resulting in the higher mortality of exposed mosquitoes.

Blood feeding percentage reduction was also high for Interceptor G2 when new, washed, and 36 months old, respectively. However, Olyset Plus was still able to reduce blood feeding to 20 and 2% when washed and at 36 months old, respectively. In this study, the penetration rate although measured, may not be appropriate for assessing the performance in terms of blood feeding inhibition and mortality due to its inherent shortcomings. While not sufficiently stipulated in the WHO guidelines, this measurement accounts for all mosquitoes found in the bait chamber, plus those blood fed in the release chamber, in calculating the penetration rate. This is an underestimation of the penetration rate because unfed mosquitoes that penetrate the net into the bait chamber and fly back through to the release chamber are not accounted for. However, one important aspect of penetration as a measure is that it gives a proxy comparison of the proportion of mosquitoes that enter the bait compared the proportion that blood feeds [32]. 

The steady decline in bio-efficacy (killing effect) of Olyset Plus nets could be attributed to the rapid loss of permethrin and PBO with time as a result of usage in the community. In previous studies, the decline in permethrin content was more rapid in Olyset ® Plus compared to Olyset ® Net [33]. Apart from that lost due to natural decay and evaporation, permethrin and PBO may be lost due to friction/abrasion, or frequent washing and drying under direct sunlight, as previously reported [34,35]. Generally, based on the laboratory results against a susceptible mosquito, only Interceptor G2 performed as it should even after 3 years (36 months) and did meet the WHO efficacy criteria of a 3-year LLIN. These results are in agreement with findings from a previous large study [20], which indicated the better overall protective efficacy of Interceptor^®^ G2 nets despite a speed decrease in a constituent active ingredient. Comparing the laboratory-washed and 36-month-old community-used nets’ effect on the bio-efficacy (mortality), the cone bioassays against a susceptible strain indicate the equivalent performance for Interceptor G2 and Olyset Plus. However, in the tunnel tests, the Olyset Plus, when laboratory-washed and community-used, showed different results in terms of mortality from the Interceptor G2. This differential performance mortality effect may be a combination of mode of action of the active ingredients, mode of net treatments (coating versus incorporation), and polymer type in the manufacturing process. Coating technologies [35], the crystal nature of the active ingredients, and the net materials used [36] have been attributed to nets’ performances in previous studies. The cone bioassays using susceptible *An. gambiae* s.s. (Kisumu) indicate significant differences in mortality rates (both 24 h mortality and 72 h mortality) for unwashed and twenty-times washed Olyset plus LLINs. However, there was no significant difference in mortality rates (both 24h mortality and 72 h mortality) for unwashed and twenty-times washed IG2 LLINs. Similar results were reported in a previous study by Tungu et al. (2021) [37], which compared the efficacy of interceptor® G2 and chlorfenapyr-treated nets where there was no significant loss in mortality with IG2 LLIN between 0 and 20 washes and, in addition, most mortality was induced by the chlorfenapyr component of IG2. In another study by Camara et al. (2018) [38], IG2 LLIN that was unwashed or washed twenty times killed 87% and 82% of *An. gambiae* s.s., respectively, whereas Interceptor LLIN, that was either unwashed or washed twenty times, killed only about 10% of the mosquitoes. The improved efficacy of twenty-times washed IG2 LLINs could be attributed to the high wash resistance of IG2 LLIN, as previously reported by N’Guessan et al. (2016) [39], where chlorfenapyr-alphacypermethrin LLINs demonstrated improved efficacy and wash resistance compared to a standard alpha-cypermethrin LN against pyrethroid-resistant mosquitoes. In the present study, lower mortalities were observed for twenty-times washed compared to unwashed Olyset LLINs. This is probably due to the loss of PBO in Olyset plus LLINs during washing. Recently, PermaNet 3.0 (deltamethrin +PBO LLIN) has shown a higher wash retention of PBO compared to Olyset Plus after twenty washes [40]

The findings from this study indicate that community usage greatly affects the physical integrity of nets compared to laboratory washing. The IG2 nets lost 54% and 40% of their bursting strength when washed in the laboratory and used in the community, respectively. No directional pattern for the physical integrity elements was observed with Olyset Plus nets in relation to laboratory wash and community usage. Also, from this study, it is noticeable that the majority of the initial and final physical strength values for the Interceptor G2 nets were relatively high compared to that of Olyset Plus, which correlates with the hole-index data from the larger study [20]. This superior physical strength suggests that the former could be more robust and likely to resist mechanical damage than the latter, something which is consistent with an earlier study This difference in physical integrity could probably be attributed to the constituent polymers of the nets; polyester type nets have been reported to be more durable than those of polyethylene [24,41]. 

The findings from our study are in line with several studies which have reported LLINs not consistently performing for the full three years in the field [42,43]. This decline in durability may impact on the gains already achieved in malaria control, if not appropriately addressed. There is a need for universal specifications that link net quality to performance, for updates to testing guidelines to reflect new products, and for building capacity for testing laboratories for quality assurance/quality control pre/post shipment and pre/post distribution [44,45]. The current proliferation of several LLIN brands in the market, although aimed at providing affordable LLINs owing to an increased demand, need to be subjected to appropriate and stringent checks to sieve out lower quality LLINs [46]. Data on LLIN durability under field conditions, that are provided by qualified testing laboratories, should serve to determine LLINs’ functional life and actual impact; they should also be used by the manufacturers to improve the quality of nets produced [45,47].

The lack of independent nets as replicates in our study is acknowledged as a limitation, although this effect was reduced by random sample selection and by the fact that all the nets used for the study belonged to the same purchase batch. Generally, nets are expected to comply to the manufacturer’s specifications (in terms of insecticide concentration and physical integrity). Hence, despite variations between individual nets within a batch, these variations are not expected to go beyond the specified range. The range specified by manufacturers for IG2 and Olyset Plus were referred to. Furthermore, there was strict adherence to the WHO procedures and set up of bioassays, exposing a large number of susceptible mosquitoes to obtain statistical significance in cone bioassay and tunnel tests, such that variations between nets are, to a large extent, attributable to net type, usage, and age. However, these steps will not completely address net variations attributable to usage habits across households and environmental conditions during field usage, which may cause differential damage to the nets, hence future studies shall consider sampling nets with different levels of damage (low, moderate, and high) after three years’ usage and assess the fabric strength and bio-efficacy.

## 5. Conclusions

This study reports on the comparative performance of nets subjected to laboratory and field usage in Tanzania through extensively checking the physical integrity and the insecticidal bio-efficacy. The results showed a better bio-efficacy for the 36-month-old Interceptor G2 nets compared to the Olyset Plus nets. However, given the reduced fabric strength for both LLINs evaluated, manufacturers should focus on improving physical integrity. Improved and more durable LLINs can be highly cost-effective when considering the reduced frequency of replacement and promoted usage. To investigate whether the observed problem is restricted to geographical setting or limited to independent sample size, we recommend that bio-efficacy and fabric integrity, in all malaria endemic countries where these nets are deployed, is conducted, and that sampling and sample size are reconsidered for sufficient study power. Our findings suggest that it might be important for the national malaria programs to consider the routine checking of nets post distribution in order to gather evidence for selecting future options for net type, in considering physical and bio-efficacy measurements as a critical step for ensuring chosen LLINs are capable of maintaining their protective efficacy throughout the defined period.

## Figures and Tables

**Table 1:** Manufacturer specifications for Olyset Plus and IG2.

Net Type	Polymer	Bursting Strength	Denier	Minimum Mesh Size (holes/cm^2^)	Active Ingredient
Olyset Plus	Polyethylene	≥250 kPa	100	6	Permethrin * 2.0% *w*/*w* (+/−0.5% *w*/*w*) Piperonyl butoxide ** 1.0% *w*/*w* (+/−0.25% *w*/*w*)
Interceptor G2	Polyester	≥405 kPa	100	24	Chlorfenapyr (200 mg/m^2^ ± 25%), alpha-cypermethrin (100 mg/m^2^ ± 25%)

* WHO specifications and evaluations for public health pesticides/40:60 *cis: trans* permethrin technical material (March 2009) ** WHO specification and evaluations for public health pesticides piperonyl butoxide https://www.who.int/whopes/quality/PBO_specs_eval_WHOSep_2011.pdf (accessed on 22 March 2023).

## Data Availability

The datasets supporting the conclusions of this article are included within the article (and its additional file(s)).

## Supplementary Materials

The following supporting information can be downloaded at: https://www.mdpi.com/article/10.3390/tropicalmed8080379/s1, Table S1: Measures of association between net treatments and mortality for susceptible *An. gambiae* Kisumu in cone bioassays; Table S2: Measures of association between net treatments and mortality for pyrethroid resistant *An. gambiae* Muleba-kis in cone bioassays; Table S3: Fabric strength results for the unwashed and washed Interceptor G2 and Olyset Plus LLINs.
