# Supplementary material for: Evaluation of Durability as a Function of Fabric Strength and Residual Bio-Efficacy for the Olyset Plus and Interceptor G2 LLINs after 3 Years of Field Use in Tanzania
Source: Trop Med Infect Dis. 2023 Jul 25;8(8):379. doi: 10.3390/tropicalmed8080379 (PMC10459516; doi:10.3390/tropicalmed8080379)
Supplement: Supplementary file 1 [file tropicalmed-08-00379-s001.zip › tropicalmed-2375273-supplementary.pdf]

Table S1: Measures of association between net treatments and mortality for susceptible *An. gambiae* Kisumu in cone bioassays

**Reference LLIN**	**Candidate LLIN**	**AOR (95% CI)**	***p*-Value**
Untreated 0x	Untreated 20x	0.29 (0.09 – 0.89)	0.031
Untreated 0x	IG2 36m	20.89 (7.72 – 56.50)	<0.001
Untreated 0x	Unused IG2 20x	11.91 (4.59 – 30.87)	<0.001
Untreated 0x	Unused IG2 0x	9.96 (3.88 – 25.62)	<0.001
Untreated 0x	Unused Olyset Plus 0x	59.69 (18.52 – 192.32)	<0.001
Untreated 0x	Unused Olyset Plus 20x	5.28 (2.08 – 13.36)	<0.001
Untreated 0x	Olyset Plus 36 months	5.28 (2.08 – 13.36)	<0.001
Untreated 20x	IG2 36m	71.83 (27.71 – 186.20)	<0.001
Untreated 20x	Unused IG2 20x	40.94 (16.51 – 101.52)	<0.001
Untreated 20x	Unused IG2 0x	34.26 (13.94 – 84.22)	<0.001
Untreated 20x	Unused Olyset Plus 0x	205.26 (66.03 – 638.04)	<0.001
Untreated 20x	Unused Olyset Plus 20x	18.14 (7.50 – 43.89)	<0.001
Untreated 20x	Olyset Plus 36 months	18.14 (7.50 – 43.89)	<0.001
IG2 36m	Unused IG2 20x	0.57 (0.27 – 1.20)	0.138
IG2 36m	Unused IG2 0x	0.48 (0.23 – 0.99)	0.048
IG2 36m	Unused Olyset Plus 0x	2.86 (1.05 – 7.81)	0.041
IG2 36m	Unused Olyset Plus 20x	0.25 (0.12 – 0.52)	<0.001
IG2 36m	Olyset Plus 36 months	0.25 (0.12 – 0.52)	<0.001
Unused IG2 20x	Unused IG2 0x	0.84 (0.43 – 1.65)	0.606
Unused IG2 20x	Unused Olyset Plus 0x	5.01 (1.91 – 13.15)	0.001
Unused IG2 20x	Unused Olyset Plus 20x	0.44 (0.23 – 0.85)	0.015
Unused IG2 20x	Olyset Plus 36 months	0.44 (0.23 – 0.85)	0.015
Unused IG2 0x	Unused Olyset Plus 0x	5.99 (2.30 – 15.59)	<0.001
Unused IG2 0x	Unused Olyset Plus 20x	0.52 (0.28 – 1.01)	0.053
Unused IG2 0x	Olyset Plus 36 months	0.52 (0.28 – 1.01)	0.053
Unused Olyset Plus 0x	Unused Olyset Plus 20x	0.09 (0.03 – 0.23)	<0.001
Unused Olyset Plus 0x	Olyset Plus 36 months	0.09 (0.03 – 0.23)	<0.001
Unused Olyset Plus 20x	Olyset Plus 36 months	1.00 (0.54 – 1.87)	1.000

Table S2: Measures of association between net treatments and mortality for pyrethroid resistant *An. gambiae* Muleba-kis in cone bioassays

**Reference LLIN**	**Candidate LLIN**	**AOR (95% CI)**	***p*-Value**
Untreated 0x	Untreated 20x	0.37 (0.11 – 1.28)	0.116
Untreated 0x	IG2 36m	1.62 (0.54 – 4.83)	0.389
Untreated 0x	Unused IG2 20x	1.49 (0.49 – 4.48)	0.480
Untreated 0x	Unused IG2 0x	5.49 (1.94 – 15.52)	0.001
Untreated 0x	Unused Olyset Plus 0x	279.84 (51.54 – 1519.49)	<0.001
Untreated 0x	Unused Olyset Plus 20x	1.00 (0.32 – 3.16)	1.000
Untreated 0x	Olyset Plus 36 months	12.50 (4.39 – 35.56)	<0.001
Untreated 20x	IG2 36m	4.40 (1.63 – 11.92)	0.004
Untreated 20x	Unused IG2 20x	4.05 (1.48 – 11.04)	0.006
Untreated 20x	Unused IG2 0x	14.95 (5.87 – 38.06)	<0.001
Untreated 20x	Unused Olyset Plus 0x	761.26 (149.08 – 3887.37)	<0.001
Untreated 20x	Unused Olyset Plus 20x	2.72 (0.95 – 7.82)	0.063
Untreated 20x	Olyset Plus 36 months	33.99 (13.24 – 87.28)	<0.001
IG2 36m	Unused IG2 20x	0.92 (0.41 – 2.06)	0.837
IG2 36m	Unused IG2 0x	3.40 (1.66 – 6.95)	0.001
IG2 36m	Unused Olyset Plus 0x	172.91 (37.99 – 787.06)	<0.001
IG2 36m	Unused Olyset Plus 20x	0.62 (0.26 – 1.48)	0.278
IG2 36m	Olyset Plus 36 months	7.72 (3.73 – 15.98)	<0.001
Unused IG2 20x	Unused IG2 0x	3.69 (1.78 – 7.66)	<0.001
Unused IG2 20x	Unused Olyset Plus 0x	188.18 (41.10 – 861.57)	<0.001
Unused IG2 20x	Unused Olyset Plus 20x	0.67 (0.28 – 1.62)	0.377
Unused IG2 20x	Olyset Plus 36 months	8.40 (4.01 – 17.60)	<0.001
Unused IG2 0x	Unused Olyset Plus 0x	50.93 (11.67 – 222.34)	<0.001
Unused IG2 0x	Unused Olyset Plus 20x	0.18 (0.08 – 0.40)	<0.001
Unused IG2 0x	Olyset Plus 36 months	2.27 (1.20 – 4.30)	0.012
Unused Olyset Plus 0x	Unused Olyset Plus 20x	0.00 (0.00 – 0.02)	<0.001
Unused Olyset Plus 0x	Olyset Plus 36 months	0.04 (0.01 – 0.20)	<0.001
Unused Olyset Plus 20x	Olyset Plus 36 months	12.50 (5.57 – 28.04)	<0.001

Table S3: Fabric strength results for the unwashed and washed Interceptor G2 and Olyset Plus LLINs

**Fabric Strength Test**		**Net Type**						**Relative Expand. Uncertainty (K=2)**
		**Interceptor G2**			**Olyset Plus**			
		**New Unwashed**	**New Washed at Laboratory 20 Times**	**36 Months Old, Washed Several Times**	**New Unwashed**	**New Washed at Laboratory 20 Times**	**36 Months Old, Washed Several Times**	
Mean bursting strength (kPa)		430	390	376	330	340	337	15%
Mean fabric weight (g/m²)		47.2	45.8	48.2	38.7	44.3	49.4	3.2%
Modified, Grab method, with hooks: Mean tensile strength (N)	Length	19	21	18	19	25	18	N/A
	Width	24	26	21	21	30	23	
Grab method, Mean tensile strength (N)	Length	224	210	196	184	182	160	16%
	Width	148	132	95.4	92.4	87.6	90.8	
Mean mesh size (holes/cm²)		31	29	30	16	21	22	N/A
Tear strength, (N)	Length	17	13.8	14.1	9.9	13.6	7.5	12%
	Width	15	19	6.9	5.0	21.4	10	

Values given represent a mean of five tests (from 5 samples) per net. N = Newton (standard unit of force)
